# Unveiling the interfacial liquid in electrochemical reactions

**DOI:** 10.1093/nsr/nwae318

**Published:** 2024-09-09

**Authors:** Joseph Nicolas, Rani Baidoun, Dohyung Kim

**Affiliations:** Department of Chemical and Biomolecular Engineering, University of Pennsylvania, USA; Department of Chemical and Biomolecular Engineering, University of Pennsylvania, USA; Department of Chemical and Biomolecular Engineering, University of Pennsylvania, USA

## Abstract

Adapting novel experimental techniques to address key knowledge gaps about the structure and properties of the interfacial liquid (IL) will enhance our understanding of its influence on electrochemical reactions, particularly in mediating species transport, charge transfer, and intermediate stability.

Initiatives to electrify the fuel and chemical industries via renewable resources have spurred large efforts to advance electrochemical systems. Despite considerable progress, these systems have yet to equal or surpass established methods. Thus far, progress has predominantly depended on the fine-tuning of electrode and catalyst surfaces. However, recent insights into the structure and dynamics of the interfacial liquid (IL) have shown promise in advancing electrocatalysis beyond existing improvements [1].

The IL is the liquid region that is in direct contact with a charged solid surface—typically an electrode or catalyst (Fig. 1A). Within this region, a double layer (DL) is formed comprising ionic species and water molecules with behavior that is distinct from that of the bulk liquid. Research has shown the significant influence of the IL on the kinetics of charge transfer, transport of species, (de)stabilization of reaction intermediates and catalyst microenvironment regulation (Fig. 1A) [1]. The structure and composition of the IL can also be influenced by the relative hydrophilicity or hydrophobicity of the solid surface, impacting these fundamental processes [1]. Its influence on important reactions such as hydrogen evolution reaction (HER), oxygen evolution and reduction reactions (OER and ORR), and carbon dioxide reduction reaction (CO_2_RR) has been well demonstrated [1]. Additionally, its properties have been shown to significantly affect the rates and selectivity of organic electrosynthesis [2,3].

**Figure 1.**
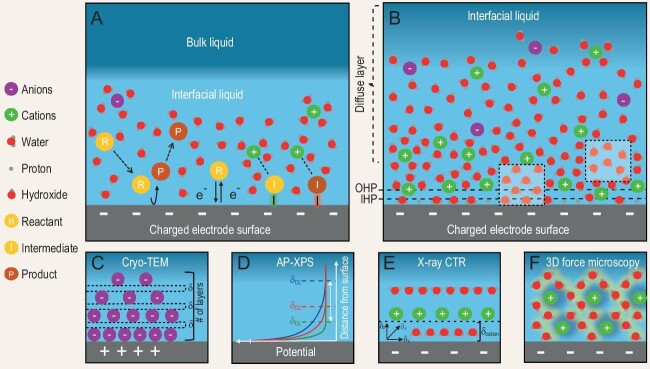
(A) Schematic of the interfacial liquid (IL) mediating species transport, charge transfer and intermediate (de)stabilization for electrocatalytic reactions (bonds highlighted in green (left) and red (right) indicate stabilization and destabilization, respectively). (B) Model of the IL based on established theoretical models. Dotted boxes highlight regions characterizable by using current spectroscopic techniques (e.g. SEIRAS, SERS and SFG). According to the Stern model, the inner Helmholtz plane (IHP), outer Helmholtz plane (OHP) and diffuse layer are annotated. (C) Visualizing ionic layering and their separation using cryo-TEM. (D) AP-XPS for measuring the potential drop across the double layer (DL), mapping its profile and the electrochemical DL depth at various electrolyte concentrations (denoted as green, red and blue from most to least concentrated). (E) Determining the 3D distribution of ions and water molecules using X-ray CTR. (F) 3D charge and water mapping using atomic force microscopy (AFM)-based techniques.

Many efforts have been made to investigate the IL or the properties of the catalytic interface resulting from a specific IL structure (Table 1). Electrochemical impedance spectroscopy has been used as a simple method to probe the interfacial properties, such as charge-transfer resistance and DL capacitance, through equivalent circuit modeling [1]. However, its effectiveness depends on accurate circuit modeling and it only provides indirect insights into molecule-level mechanisms. Direct probing techniques such as surface-enhanced infrared absorption spectroscopy (SEIRAS) and

surface-enhanced Raman spectroscopy (SERS) have been used to study the degree of structural (dis)order of water molecules (Fig. 1B) [1]. More sophisticated and interface-selective non-linear optical techniques such as sum frequency generation (SFG) have been used to probe the alignment and orientation of water molecules (Fig. 1B) [4]. However, SEIRAS, SERS and SFG are more suited to investigating organic adsorbates on catalyst surfaces and they lack sufficient capacity to probe the density and distribution of ionic species (e.g. alkali-metal cations) within the IL, which are its critical components (Table 1) [5,6].

**Table 1.** Summary of the experimental methods currently used to probe the interface or the IL in electrocatalytic systems.

Technique	Capabilities	Limitations
Electrochemical impedance spectroscopy	Combined with equivalent circuit modeling, offers straightforward access to key properties of a catalytic interface under operando conditions, such as charge-transfer resistance, double-layer capacitance and mass transport resistance	Primarily provides indirect insights and can be prone to ambiguities in model selection and interpretation
Surface-enhanced infrared adsorption spectroscopy and surface-enhanced Raman spectroscopy	Strong signals from molecules adsorbed on or near the surface of electrodes and catalysts. Offers direct insights from the vibrational modes of the molecules present at the interface. SEIRAS exhibits higher S/N ratios and has a wider applicability for various metals. SERS can extend below 1000 cm^−1^ to probe metal–organic (e.g. C, O) vibrations originating from surface-bonded species	Not suitable for probing certain ionic species (e.g. metal cations) lacking vibrational modes. Lacking spatial (i.e. vertical and lateral) information. Precise quantification of detected species is challenging
Sum frequency generation	Inherently interface sensitive (because of the non-centrosymmetric environment requirement) with minimal interference from the bulk solution. More detailed information on the orientation and arrangement of molecules at the solid–liquid interface	Complexity in terms of instrumentation required and the interpretation of the spectra. Suffers from similar issues to other vibrational spectroscopy in not being able to probe species lacking vibrational modes. Lacking spatial information as well as challenges associated with quantification

Most of our current knowledge about the IL structure is based on theoretical approaches such as continuum models (e.g. Gouy–Chapman–Stern model) and *ab initio* molecular dynamics (Fig. 1B) [7]. Although these models offer a helpful depiction of the constituents of the DL and water molecules in the IL, they still face limitations in fully capturing the realities of the IL. For instance, molecule-level interactions, such as hydrogen bonding, are often overlooked in such continuum models [4,7]. Furthermore, the compositions and sizes of electrochemical interfaces are often oversimplified due to high computational costs [7]. There are continued efforts to bridge the gaps by combining *ab initio* methods with macroscale mean-field modeling.

There is a need for experimental methods that can precisely and quantitatively analyse different components of the IL, providing results that can even inform or be compared in depth with theoretical models [8]. As described earlier, existing methods that are commonly employed are more focused on analysing surface adsorbates and reaction intermediates but provide little information about the IL itself. In this perspective, we highlight the methods that are traditionally used in other fields or for other purposes that could potentially provide valuable information and address gaps that are present in current experimental approaches used for studying the IL (Table 2 and Fig. 1C–F). By further developing the undermentioned characterization techniques for use in common electrochemical systems, we can enhance our understanding of the structural details of the IL through the direct probing of species (ions, water molecules), their structuring and the resulting charge gradients within the IL and their impact on the electrochemical reactions.

**Table 2.** A summary of potentially useful techniques for studying the IL structure discussed in this perspective.

Technique	System	Demonstrated capabilities	Limitations
Cryo-TEM [9]	Gold nanorod/phosphotungstic acid (H_3_PW_12_O_40_)	Imaging large ion layering near the surface of a gold nanorod	Limited resolution to large ions (∼0.7 nm) because of the sensitivity of vitrified water to an electron beam
Ambient pressure X-ray photoelectron spectroscopy [10]	Gold polycrystalline electrode/KOH and pyrazine solution	Measuring the potential distribution across the double layer using a probe molecule	Restricted in terms of the electrochemical environment that can be applied (e.g. thin liquid layer geometry, electrolyte concentration)
X-ray crystal truncation rod [11]	Pt(111) electrode/0.1 M CsF	Capturing ion and water ordering and structure in the Stern layer with specific distance values	Limited to high-quality and well-ordered surfaces
Charge profiling 3D atomic force microscopy [12]	Highly oriented pyrolytic graphite (HOPG)/methylimidazolium bis(trifluoromethyl-sulfonyl)imide, LiTFSI	Angstrom-scale profiling of charge density distribution in the electric double layer for ionic liquids and water-in-salts on HOPG	Developments needed to resolve more complex electrolytes containing multiple charged species and neutral molecules (e.g. H_2_O)
3D fast force microscopy [13]	AlOOH (Boehmite) (010)/NaOH solution	Measuring water structure at the surface of hydrated minerals at sub-nm resolution	The results highly depend on tip geometry, chemistry and confinement

Cryo-electron microscopy—a technique originally used to image biological samples—was recently demonstrated to successfully image individual ions and their distributions near the surface of nanoscale samples. Li *et al*. revealed the structuring and layering of multivalent Keggin anions on the surface of Au nanorods at the single-ion level (Fig. 1C) [9]. This is an exciting development considering the lack of direct observational techniques for ions at the solid–liquid interface. However, there are limitations to applying cryo-TEM to electrochemical interfaces and ILs at this stage. Specifically, cryo-TEM has a weak ability to resolve smaller ions with low electron density through a vitrified water layer. This technique, further developed for lighter elements and higher resolutions, could help provide insights into single-ion distributions in the IL.

Another technique that could be adapted for studying the IL is ambient pressure X-ray photoelectron spectroscopy (AP-XPS), as demonstrated by Favaro *et al*. [10]. By following the spectral broadening of oxygen in water and nitrogen in pyrazine against an Au electrode, they managed to measure the potential drop and its profile across the DL, in addition to variations related to the applied potential and the electrolyte concentration (Fig. 1D). This method is especially valuable for directly estimating electric field strengths—key to charge-transfer reactions across solid–liquid interfaces—without having to infer from simplified IL models. Despite its uniqueness, the method is restricted to certain environments such as low electrolyte concentrations (<1 mM) with long screening lengths (∼10 nm) and, thus, improvements are necessary to advance this technique for more realistic electrochemical conditions.

To resolve the layering of both counterions and water at the electrode surface, Liu *et al*. demonstrated using X-ray crystal truncation rods (CTRs) [11]. They obtained high-precision data which showed that, at negative potentials, the Stern layer comprises one cation layer and two layers of water (Fig. 1E). Specifically, the cations form a sublattice structure intercalated by water. One of the water layers lies between the cations and the electrode surface whereas the second water layer acts as a hydrating layer between the Stern and diffuse layers. Furthermore, they were able to estimate the precise distance between the cation layer and the metal surface. While this specific structure cannot be directly extrapolated to other types of metal or metal-oxide surfaces due to their varying capacities for interacting with ions or water molecules [14], the insights gained offer valuable guidance for molecule-level expectations. Applying CTRs to other model surfaces will deepen our understanding of the IL, highlighting both the fundamental commonalities and the differences in ion and water structuring.

Atomic force microscopy (AFM) is typically used to study material surfaces and can also be adapted to directly probe critical aspects of the IL, such as the spatial variations of ionic species. Bonagiri *et al*. utilized charge profiling 3D AFM (CP-3D-AFM) to experimentally determine the real-space charge distribution with angstrom-level resolution (Fig. 1F) [12]. They probed charge rearrangements in highly concentrated water-in-salt electrolytes and ionic liquids, finding that the first charged layer in the IL overbalanced the surface charge of the electrode, i.e. overscreening. The advancement of similar techniques to enable charge distribution mapping in less concentrated electrolytes containing neutral species (e.g. H_2_O) could pave the way for a deeper understanding of the IL for electrocatalytic systems. Another AFM-based technique is 3D fast force mapping (3D-FFM), which, instead of probing the charge and potential distribution similarly to the CP-3D-AFM, has an oscillating tip that creates a 3D force map that can be interpreted as variations in the surface hydration layers. It was recently demonstrated to probe the laterally structured water layers near (<1 nm) mineral surfaces (Fig. 1F) [13]. The adaptation of this method to electrocatalyst surfaces could offer deeper insights into the structure of nearby water molecules and their influence on electrochemical reactions.

To develop a more complete picture of the IL, we need to combine a multitude of techniques that provide direct atomistic and molecule-level information. The techniques discussed in this perspective, which are traditionally used to investigate biological samples and obtain chemical and structural information on material surfaces, have recently shown potential for the study of ions and water molecules near the surface of various materials under restricted conditions. Given the current lack of direct methods for investigating the IL structure, despite its importance for electrocatalytic reactions, these techniques, if properly developed, could provide a comprehensive understanding of the IL and its influence on reactions.

For instance, how alkali metals are physically distributed in terms of their distance from the surface and local concentration, as well as their chemical nature in terms of the surrounding water molecules and the resulting likelihood of their specific adsorption, are all crucial pieces of information for strengthening our understanding of cation effects for the CO_2_ reduction reaction. Although the general trends amongst the Group 1 metals are known, there remain controversies as to the fundamental mechanism by which these cations enhance CO_2_ reduction, whether it is due to indirect electric field effects or direct local interactions between the cation and intermediates that require cation dehydration [1,15].

Furthermore, the techniques discussed should apply to both cathodic and anodic reactions, as they are direct physical probing methods for the IL, independently of reactive species. This broadens our understanding of the IL, especially for anodic reactions in which the interface involves a positively charged electrode with anions as primary components of the DL. While much of the interest has been in the IL for cathodic reactions (e.g. HER, CO_2_RR), similar phenomena—such as the intermediate stabilization and regulation of species transport and charge transfer—are likely to occur in anodic reactions as well. Anions have often been viewed as surface-poisoning agents in certain cases (e.g. OER/ORR) [1], yet their roles in less-studied reactions, such as partial methane oxidation, in which chloride ions were recently shown to act as promoters [16], warrant further exploration. Additionally, the influence of the IL on the anodic stability of metal and metal-oxide electrocatalysts, which is critical for durability, is another area in which our understanding lags. As the dissolution of metal ions in the IL matrix is driven by their energetic favorability (e.g. stabilization by complexation with anions and water molecules), an understanding of this process could reveal strategies for modifying the IL structure, enhancing durability without significantly compromising activity. Mastering our understanding of the IL is a crucial stepping stone toward elevating electrochemical processes across a broad spectrum of sustainable applications.
